# HLA-C KIR-Ligands Determine the Impact of Anti-Thymocyte Globulin (ATG) on Graft versus Host and Graft versus Leukemia Effects Following Hematopoietic Stem Cell Transplantation

**DOI:** 10.3390/biomedicines5020013

**Published:** 2017-03-28

**Authors:** Johannes Clausen, Alexandra Böhm, Irene Straßl, Olga Stiefel, Veronika Buxhofer-Ausch, Sigrid Machherndl-Spandl, Josef König, Stefan Schmidt, Hansjörg Steitzer, Martin Danzer, Hedwig Kasparu, Ansgar Weltermann, David Nachbaur

**Affiliations:** 1 Department of Hematology and Oncology, Elisabethinen Hospital, 4020 Linz, Austria; alexaboehm@yahoo.de (A.B.); irene.strassl@ordensklinikum.at (I.S.); olga.stiefel@ordensklinikum.at (O.S.); veronika.buxhofer-ausch@ordensklinikum.at (V.B.-A.); sigrid.machherndl-spandl@ordensklinikum.at (S.M.-S.); josef.koenig@ordensklinikum.at (J.K.); hedwig.kasparu@ordensklinikum.at (H.K.); ansgar.weltermann@ordensklinikum.at (A.W.); 2 Department of Hematology and Oncology, Medical University, 6020 Innsbruck, Austria; stefan.schmidt@i-med.ac.at (S.S.); david.nachbaur@i-med.ac.at (D.N.); 3 Austrian Red Cross, Transfusion Service for Upper Austria, 4020 Linz, Austria; hansjoerg.steitzer@o.roteskreuz.at (H.S.); martin.danzer@o.roteskreuz.at (M.D.)

**Keywords:** hematopoietic stem cell transplantation, serotherapy, antithymocyte globulin, graft-versus-host disease, HLA-C, killer cell immunoglobulin-like receptor, KIR ligand

## Abstract

Rabbit anti-thymocyte globulins (ATGs) are widely used for the prevention of acute and chronic graft versus host disease (aGVHD, cGVHD) following allogeneic hematopoietic stem cell transplantation (HSCT). However, most prospective and retrospective studies did not reveal an overall survival (OS) benefit associated with ATG. Homozygosity for human leukocyte antigen (HLA)-C group 1 killer-cell immunoglobulin-like receptor ligands (KIR-L), i.e. C1/1 KIR-L status, was recently shown to be a risk factor for severe aGVHD. Congruously, we have previously reported favorable outcomes in C1/1 recipients after ATG-based transplants in a monocentric analysis. Here, within an extended cohort, we test the hypothesis that incorporation of ATG for GVHD prophylaxis may improve survival particularly in HSCT recipients with at least one C1 KIR-ligand. Retrospectively, 775 consecutive allogeneic (excluding haploidentical) HSCTs were analyzed, including peripheral blood and bone marrow grafts for adults with hematological diseases at two Austrian HSCT centers. ATG-Fresenius/Grafalon, Thymoglobuline, and alemtuzumab were applied in 256, 87, and 7 transplants, respectively (subsequently summarized as “ATG”), while 425 HSCT were performed without ATG. Median follow-up of surviving patients is 48 months. Adjusted for age, disease-risk, HLA-match, donor and graft type, sex match, cytomegalovirus serostatus, conditioning intensity, and type of post-grafting GVHD prophylaxis, Cox regression analysis of the entire cohort (*n =* 775) revealed a significant association of ATG with decreased non-relapse mortality (NRM) (risk ratio (RR), 0.57; *p =* 0.001), and overall mortality (RR, 0.71; *p =* 0.014). Upon stratification for HLA-C KIR-L, the greatest benefit for ATG emerged in C1/1 recipients (*n =* 291), by reduction of non-relapse (RR, 0.34; *p =* 0.0002) and overall mortality (RR, 0.50; *p =* 0.003). Less pronounced, ATG decreased NRM (RR, 0.60; *p =* 0.036) in HLA-C group 1/2 recipients (*n =* 364), without significantly influencing overall mortality (RR, 0.70; *p =* 0.065). After exclusion of higher-dose ATG-based transplants, serotherapy significantly improved both NRM (RR, 0.54; *p =* 0.019; *n =* 322) and overall mortality (RR, 0.60; *p =* 0.018) in C1/2 recipients as well. In both, C1/1 (RR, 1.70; *p =* 0.10) and particularly in C1/2 recipients (RR, 0.94; *p =* 0.81), there was no statistically significant impact of ATG on relapse incidence. By contrast, in C2/2 recipients (*n =* 121), ATG neither reduced NRM (RR, 1.10; *p =* 0.82) nor overall mortality (RR, 1.50; *p =* 0.17), but increased the risk for relapse (RR, 4.38; *p =* 0.02). These retrospective findings suggest ATG may provide a survival benefit in recipients with at least one C1 group KIR-L, by reducing NRM without significantly increasing the relapse risk.

## 1. Introduction

Serotherapy using one of the two commercially available rabbit anti-thymocyte globulin (ATG) preparations is commonly applied for in vivo T cell depletion in allogeneic hematopoietic cell transplantation (HSCT), with the aim to augment standard prophylaxis against graft versus host disease (GVHD). Prospective and retrospective studies have demonstrated a significant potential of ATG to reduce the incidence and severity of acute and chronic graft versus host disease (GVHD). However, as recently reviewed [1], most of these studies failed to show a survival benefit resulting from the improved control of GVHD. This may in part be due to the studies’ population size and follow-up period, since prevention of late-onset non-relapse mortality (NRM) due to steroid-refractory or -dependent cGVHD and resulting infections, may show its benefit as late as several years after HSCT [2,3,4,5]. On the other hand, the advantage of diminished GVHD may be outweighed in part by an increased relapse rate, particularly in the case of higher ATG doses [6,7]. Therefore, most centers attempt to restrict the use of ATG to transplants with an increased risk for developing severe GVHD.

Several risk factors for severe acute and/or chronic GVHD have been established, such as an incomplete human leukocyte antigen (HLA) match, unrelated donor, G-CSF mobilized peripheral blood (PB) stem cells, sex mismatched female donor, and age of recipient and donor. Despite this, the occurrence of severe GVHD, particularly severe aGVHD, is hardly predictable in an individual transplant. Even less, the development of steroid-refractory aGVHD can be accurately predicted at the time of HSCT, although sex mismatched female donor, unrelated donor, and absence of ATG have been described as risk factors [8]. Therefore, more precise estimation of the individual GVHD risk will likely help improve algorithms on when and how to intensify GVHD prophylaxis by incorporation of ATG.

Besides many others, as recently summarized [9,10,11], we have previously suggested a role for the HLA class I ligands to inhibitory killer cell immunoglobulin-like receptors (KIR) in transplant outcomes [12]. In particular, HLA-C KIR ligands (KIR-L), which can be divided into a C1 and C2 group according to their respective inhibitory KIR, may influence the risk for both relapse and GVHD. Recipient homozygosity for one HLA-C KIR-L group, particularly the C1 group, has repeatedly been shown to increase the risk for severe aGVHD [13,14,15,16,17,18]. In addition, we have previously shown that in peripheral blood stem cell transplants (PBSCT) from HLA matched siblings applying standard GVHD prophylaxis without ATG, C1/2 recipients had superior outcomes [19], whereas after PBSCT utilizing ATG for intensified GVHD prophylaxis, recipients with C1/1 KIR-L status had improved outcomes due to a low relapse rate compared to recipients with at least one C2 allele [20]. The aim of the present study was therefore to validate in a multicenter setting the association of HLA-C1 group KIR-L with major HSCT outcomes, and to assess the suitability of different dose levels of ATG to overcome the putative GVHD risk factor, C1 KIR-L homozygosity, after HSCT.

## 2. Patients and Methods

To address the impact of ATG use and dosage on overall and non-relapse mortality, and the incidence of aGVHD, cGVHD, and relapse, within strata defined by recipient HLA-C KIR ligand status, data from 775 consecutive, allogeneic (fully or partially HLA-matched) HSCT for hematological diseases in adult patients at two Austrian EBMT centers (EBMT-CIC 271-Innsbruck, and EBMT-CIC 594-Linz) were retrospectively analyzed. Transplants were performed between 1988 and 2015, with the majority of transplants (83%) performed later than 1 January 2000. Graft source was G-CSF-mobilized PB in 632, and bone marrow (BM) in 143 cases. Excluded from the analysis are ex vivo T cell depleted grafts, cord blood, and haploidentical-related grafts using post-transplant cyclophosphamide. In accordance with the Declaration of Helsinki, all patients provided written informed consent to the treatment, and to data collection and analysis.

The major patient, disease, and transplant details are shown in Table 1. The indicated ATG doses refer to the total dose given (per kg of bodyweight), which was typically applied in two to three single doses, with the last infusion on day 1. Antiallergic and antipyretic pre- and co-medication consisted of high-dose steroids (2 × 250 mg prednisolone or 2 × 20 mg dexamethasone), intravenous antihistaminic, and, in CIC 594, also included 1 g methamizole. ATG was infused over 12 h (CIC 274, predominantly using Thymoglobuline) or over 6 hours each day (CIC 594, for ATG-F, currently starting with a maximum of 8 mg/kg fraction on the first day) with continuous monitoring of circulatory and respiratory functions. The predominant indication for the use of ATG was an unrelated transplant. However, ATG was also used in a substantial proportion of matched related transplants (e.g., per protocol in the setting of the “FLAMSA-RIC” sequential conditioning for active leukemia), besides its use in non-malignant diseases. From 2013 on, ATG was additionally used in the majority of matched related PBSCT in CIC 594.

## 3. Definitions and Grading

KIR-L group affiliation of each HLA-C allele was determined using an online database [21].

Overall survival (OS) is defined as survival time from transplant (i.e., the first day of stem cell infusion); progression-free survival (PFS) as time from transplant to progression/relapse or death; non-relapse mortality (NRM) as mortality without previous or ongoing progression or relapse; GVHD-associated NRM as any NRM in a patient with previous or ongoing aGVHD grade II–IV. Acute GVHD was graded according to Glucksberg or modified Glucksberg criteria [22]. Chronic GVHD was either graded as limited or extensive according to the original Seattle criteria, or, from the mid-nineties on, as mild, moderate, or severe, according to the NIH consensus [23]. The definition of myeloablative versus reduced intensity conditioning was applied as previously proposed [24]. Disease stage was categorized as either early/low-risk (i.e., malignancy in first complete remission/CR, low-risk MDS or MPN, or non-malignant disease) or intermediate or advanced/high-risk (intermediate = second CR, intermediate-risk MDS or MPN; advanced/high-risk = active disease at the time of transplant or high/very-high-risk MDS or MPN, or relapse of malignancy after a previous allogeneic HSCT).

## 4. Statistical Methods

Statistical analyses were performed using NCSS 2001 software (NCSS, Kaysville, UT, USA). For a univariate analyses, OS and PFS probabilities were estimated using Kaplan-Meier statistics. Survival curves were compared using the log-rank test. Two-sided *p*-values < 0.05 were considered significant. Cumulative incidences for relapse/progression, NRM, aGVHD, and cGVHD were calculated considering the respective competing risks (i.e., NRM for relapse/progression, relapse/progression for NRM, and death without aGVHD/cGVHD for aGVHD/cGVHD, respectively). For multivariate analyses the Cox proportional hazards model was applied. The following variables were included: age of donor and recipient as continuous variables, and the categorical variables, ATG-treatment (yes vs. no), disease stage (early vs. intermediate/advanced), graft source (BM vs. G-CSF mobilized PB), HLA mismatch (vs. 10/10 match), recipient/donor relationship (related vs. unrelated), sex mismatch (female to male vs. other), cytomegalovirus (CMV) serostatus of recipient and donor, conditioning intensity (myeloablative vs. reduced intensity or non-myeloablative), and type of post-grafting GVHD prophylaxis (methotrexate-based vs. other). Variables without significant impact were stepwise excluded from the model until only those with a *p*-value ≤0.2 remained. Only those variables with a *p*-value ≤0.05 are shown in the results tables, except for the effect of ATG, which is always indicated irrespective of its significance level. Analyses were performed either for all ATG dose levels (i.e., no ATG vs. ATG at any dose), or only the lower ATG dose levels. Lower-dose ATG was defined as ATG-Fresenius (ATG-F) ≤30 mg/kg or Thymoglobuline ≤5 mg/kg. Alemtuzumab (median dose 50 mg flat (40 mg–100 mg) was always considered as high dose serotherapy. To investigate the effect of ATG in a more homogenous setting with regard to transplant indication and type, additional analyses were performed in a cohort restricted to HLA-matched PBSCT for malignant diseases (*n =* 507).

## 5. Results

### 5.1. Impact of ATG on Overall Survival—Entire Cohort

**Figure 1:**
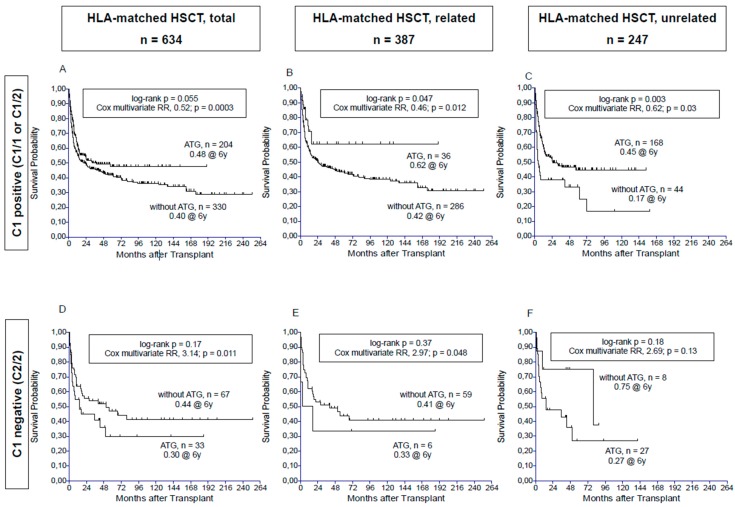
Probability of overall survival after HLA-matched transplants, according to the use of ATG, for the C1 positive (C1/1 or C1/2) KIR-L cohort (**A**–**C**) and for the C2/2 cohort (**D**–**F**). Figure 1**A**,**D** show the Kaplan-Meier curves for the entire cohorts, Figure 1**B**,**E** for related, and Figure 1**C**,**E** for unrelated transplants. The indicated log-rank *p*-values refer to the univariate comparison of the ATG versus no-ATG curves, and impact of ATG within the respective Cox multivariable model is also indicated.

In the overall cohort including recipients of fully or partially HLA-matched (excluding haploidentical) allogeneic peripheral blood and marrow transplants (*n =* 775), Cox regression multivariable analysis revealed an association of ATG with decreased overall mortality (Table 2). If transplants with higher-dose ATG (i.e., ATG-F ≥35 mg/kg, Thymoglobuline ≥7.5 mg/kg, or Alemtuzumab 40–100 mg) were excluded from the analysis, the remaining lower-dose ATG based transplants (ATG-F ≤30 mg/kg or Thymoglobuline ≤5 mg/kg) were associated with an even more pronounced survival benefit (Table 2). In recipients homozygous for HLA-C1 group KIR ligands (C1/1), ATG provided a significant survival benefit at all dose levels, and even more at the lower dose level (Table 2). In recipients with C1/2 group KIR-ligands, ATG was associated with improved survival only at the lower dose level, but not if the higher doses were included. As shown in Figure 1A, in recipients of HLA-matched HSCT with at least one C1 group allele (i.e., C1/1 or C1/2; *n =* 634), ATG was associated with a non-significant survival advantage in the unadjusted univariate analysis, but with a highly significant advantage (RR, 0.52; *p =* 0.0003) in multivariate analysis, adjusting for the most relevant confounding factor, unrelated donor. Accordingly, separate analyses by donor type of HLA-matched HSCT revealed a significant benefit for ATG in both related and unrelated transplants in the C1/x KIR-L group. This applies to both univariate and multivariate testing (Figure 1B,C).

In contrast to the C1/1 and the C1/2 group, ATG provided no survival benefit in C2 homozygous recipients, irrespective of its dose level (Table 2). Also, after exclusion of HLA-mismatched transplants in the C2/2 group, ATG-based HSCT had inferior survival outcomes by multivariate analysis than those without ATG, independent of the donor-recipient relationship (Figure 1D,F).

### 5.2. Impact of ATG on Overall Survival—HLA-Matched PBSCT in Malignant Diseases

Next, the analysis was restricted to HLA-matched peripheral blood transplants for malignant diseases, since, despite a multivariable testing, the above findings may be biased by the disequilibrium of ATG utilization and dosage in favor of non-malignant disease. Furthermore, it cannot be excluded that the above findings are influenced by a particular benefit of ATG in HLA-mismatched HSCT. Finally, bone marrow transplants (BMT) were excluded to yield a more homogenous treatment setting, and because the number of BMT was too small to show (or exclude) an association of the effects of ATG with the HLA-C KIR-L status (data not shown). Within the remaining cohort (i.e., HLA-matched PBSCT for hematological malignancies (*n =* 507)), ATG at all dose levels had no significant impact on overall mortality, while after restriction to the lower dose levels, ATG was significantly associated with reduced overall mortality (Table 3). Stratification by HLA-C KIR-L status revealed a significant association of ATG with decreased mortality within the C1/1 KIR-L group, both at all dose levels, and at the lower ATG dose levels (Table 3). In C1/2 recipients, ATG at neither all dose levels nor the lower doses provided a significant survival benefit (Table 3). However, in the combined cohort represented by recipients with at least one C1 KIR-L (i.e., C1/1 or C1/2) receiving HLA-matched PBSCT for malignant disease, ATG was significantly associated with decreased mortality, both at all dose levels (RR, 0.62; *p =* 0.01; *n =* 441), and even more at the lower dose levels (RR, 0.50; *p =* 0.002, *n =* 391). By contrast, in recipients homozygous for C2 KIR-L, ATG was associated with a trend toward increased overall mortality (Table 3).

### 5.3. Impact of ATG on Non-Relapse Mortality

Overall, ATG significantly reduced the risk for non-relapse mortality by a multivariable analysis (RR, 0.57; *p =* 0.001; *n =* 775). Stratification of the entire cohort for HLA-C KIR-L status revealed a highly significant, 3-fold reduction of NRM within the C1/1 group by ATG at all dose levels (RR, 0.33; *p =* 0.0001; *n =* 291), and similarly, at the lower dose levels (RR, 0.31; *p =* 0.0004; *n =* 235). In C1/2 recipients, reduction of NRM by ATG was less pronounced, yet marginally significant, if all ATG doses were included (RR, 0.61; *p =* 0.04; *n =* 363), and was slightly more pronounced if only the lower ATG dose levels were considered (RR, 0.56; *p =* 0.026; *n =* 320). In C2/2 recipients, there was no significant effect of ATG on NRM, irrespective of whether all (RR, 1.13, *p =* 0.8; *n =* 121) or only the lower ATG doses were considered (RR, 0.70; *p =* 0.5; *n =* 101).

After restriction to HLA-matched PBSCT for malignant diseases, the effect of ATG on NRM was similar to that in the entire cohort (RR, 0.50; *p =* 0.005; *n =* 507). Likewise, in transplants utilizing lower-dose ATG within this cohort, the benefit of ATG was similarly pronounced (RR, 0.47, *p =* 0.005; *n =* 449).

### 5.4. Impact of ATG on aGVHD Grade 3–4

The cumulative incidence of aGVHD grade 3–4 (at one year post-HSCT) among patients not receiving ATG prophylaxis was 27.1%, with the highest incidence in the C1/1 subgroup (30.6%), followed by the C1/2 (26.4%), and the C2/2 group (21.1%; Table 4). Among all ATG-treated patients, the one-year cumulative incidence of aGVHD 3-4 reached 22.6% (Table 4). By multivariable analysis adjusting for the higher proportion of unrelated and mismatched transplants in the ATG cohort, serotherapy was significantly associated with a reduced incidence of aGVHD grade 3–4 (RR, 0.63; *p =* 0.02; *n =* 775). After restriction to lower-dose ATG, this effect was no longer significant (RR, 0.70; *p =* 0.1; *n =* 656).

Stratification by HLA-C KIR-L status revealed a significant protective effect of ATG against aGVHD 3–4 within the C1/1 cohort (RR, 0.44; *p =* 0.01; *n =* 291), but not in the C1/2 or the C2/2 cohort (Table 4). Consequently, among ATG-treated patients the incidences of aGVHD 3–4 were nearly identical in the C1/1 compared to the C1/2 and the C2/2 group (Table 4), suggesting ATG may overcome the risk for severe aGVHD associated with C1 homozygosity. Addressing this hypothesis in more detail, the association of C1 homozygosity with aGVHD 3–4 was separately assessed in non-ATG treated and ATG treated PBSCT recipients, respectively. In patients who did not receive ATG prior to PBSCT (*n =* 331), the impact of C1 homozygosity on aGVHD 3–4 was of a similar magnitude (RR, 1.94; *p =* 0.09) as was HLA-mismatch (RR, 2.06; *p =* 0.046) and sex mismatched female donor (RR, 1.83; *p =* 0.01). In ATG-treated PBSC recipients (*n =* 301), however, C1 homozygosity entirely lost its impact on the risk for aGVHD 3–4 (RR, 0.81; *p =* 0.58), as did a sex mismatched female donor (RR, 0.83; *p =* 0.51). In contrast, an HLA-mismatch remained a highly significant risk factor for aGVHD 3–4 even in ATG-based PBSCT (RR 2.55; *p =* 0.0002).

### 5.5. Impact of ATG on aGVHD-Associated NRM

The incidence of aGVHD-associated NRM at two years post-HSCT, defined as NRM in any patient with previous or ongoing aGVHD requiring systemic treatment (i.e., grade 2–4) was 24.9% after transplants without ATG, and was 20.5% after those with ATG (Table 4). Similar to aGVHD 3–4, the highest incidence of aGVHD-associated NRM after non-ATG based transplants was found in the C1/1, and the lowest in the C2/2 group (Table 4). Despite the apparently marginal difference in the univariate comparison of ATG-based and non-ATG-based HSCT, multivariate analysis revealed that ATG was significantly associated with a reduced incidence of aGVHD-associated NRM within the entire cohort (RR, 0.51; *p =* 0.001; *n =* 775). Stratification by KIR-L status revealed that this effect was most prominent in the C1/1 KIR-L group (RR, 0.28; *p =* 0.0002; *n =* 291), while being of borderline significance in the C1/2 KIR-L group (RR, 0.55; *p =* 0.04; *n =* 363). By contrast, in recipients with C2/2 KIR-ligands, ATG obviously did not at all reduce the incidence of aGVHD-associated NRM (RR, 1.34; *p =* 0.6; *n =* 121). Consequently, in ATG-treated recipients the incidence of aGVHD-associated NRM was lowest in the C1/1 and highest in the C2/2 cohort, showing a reverse order compared to non-ATG-treated HSCT recipients (Table 4).

### 5.6. Impact of ATG on cGVHD

Overall, the use of ATG was significantly associated with a reduced incidence of moderate/severe or extensive cGVHD by multivariate Cox regression analysis (RR, 0.27; *p <* 0.0001; *n =* 775). If only the lower ATG dose levels were considered, moderate/severe or extensive cGVHD was decreased at nearly the same extent (RR, 0.31; *p <* 0.0001; *n =* 656). Stratification by KIR-L status revealed that ATG was significantly associated with a reduced incidence of moderate/severe or extensive cGVHD in the C1/1 cohort (RR, 0.45; *p =* 0.018; *n =* 291), but had no significant impact if only the lower ATG dose levels were considered (RR, 0.58; *p =* 0.13; *n =* 235). More profoundly, ATG reduced moderate/severe cGVHD within the C1/2 group under consideration of all dose levels (RR, 0.17; *p <* 0.0001; *n =* 363), and even if only the lower ATG dose levels were considered (RR, 0.18; *p <* 0.0001; *n =* 320). A similar reduction of moderate/severe or extensive cGVHD was found in the C2/2 group by ATG at all dose levels (RR, 0.25; *p =* 0.005; *n =* 121) and, as a trend, by the lower ATG dose levels (RR, 0.33; *p =* 0.06; *n =* 101).

### 5.7. Impact of ATG on Relapse

For assessing the impact of ATG on the relapse risk, only transplants for malignant diseases were considered (*n =* 749). In this cohort, ATG did not significantly increase the risk for relapse by multivariate analysis, both under consideration of all ATG dose levels (RR, 1.27; *p =* 0.22) or only the lower dose levels (RR, 1.15; *p =* 0.52; *n =* 642). Upon stratification for HLA-C KIR-L status, ATG did not significantly increase the relapse risk within the C1/1 cohort (RR, 1.47; *p =* 0.22; *n =* 271, including all ATG dose levels, and RR, 1.28; *p =* 0.48; *n =* 225, including only lower-dose ATG). After further restriction to HLA-matched PBSCT, ATG appeared to increase the relapse risk in the C1/1 cohort particularly if the higher ATG dose levels were included (RR, 1.70; *p =* 0.10; *n =* 185), but less so upon restriction to the lower ATG dose levels (RR, 1.46; *p =* 0.3; *n =* 155). By contrast, in C1/2 recipients, there was not even a trend towards an increased relapse risk in patients treated with ATG, irrespective of whether all ATG dose levels were included (RR, 0.94; *p =* 0.81; *n =* 356), or the lower ATG dose levels only (RR, 0.89; *p =* 0.66; *n =* 317). Restriction of the analysis to HLA-matched PBSCT did not alter the findings in C1/2 recipients (data not shown). In the C2/2 KIR-L cohort, however, ATG significantly increased the relapse risk (RR, 4.38; *p =* 0.02; *n =* 115).

## 6. Discussion

This retrospective study of a large, two-center HSCT cohort is the first one addressing the differential impact of anti-T cell serotherapy on HSCT outcomes after stratification for HLA-C KIR-L status. The study reveals three major findings which will be discussed below: (a) It confirms the previously reported significance of an HLA-C1 homozygous (C1/1) KIR-L status as risk factor for severe aGVHD and NRM; (b) Findings further suggest that serotherapy with ATG is appropriate to overcome the aGVHD risk factor, C1 homozygosity, and even more, to limit aGVHD-associated NRM particularly in this high-risk group; (c) Finally, we show here for the first time differential effects of ATG on aGVHD, cGVHD, relapse, and NRM, in the C1/1, the C1/2, and the C2/2 recipient cohorts, respectively. Most important among them is the novel observation of a highly efficient control of cGVHD by ATG in C1/2 heterozygous recipients without any impact on their relapse risk.

Homozygosity for HLA-C1 KIR-ligands in the recipient has previously been shown to increase the risk for severe aGVHD [13,14,15,16,17,18]. Although readily available upon HLA-C high-resolution typing without the need for KIR genotyping of donor or recipient, this risk factor has never been validated in a prospective fashion and has hardly gained attention in clinical practice. The present findings clearly support the above studies, and beyond this, they reveal C1 KIR-L homozygous recipients to be at high risk for aGVHD-associated NRM if they were not pretreated with ATG.

The novel finding that serotherapy with ATG may entirely overcome the adverse impact of C1 KIR-L homozygosity on severe aGVHD and on NRM appears to be clinically relevant. Interestingly, while ATG similarly eliminated another aGVHD risk factor in the present study (i.e., female donor to male recipient), an HLA mismatch still doubled the risk for severe aGVHD in ATG-based transplants, as it did in non-ATG-based ones. Thus, HLA-C1/1 KIR-L status and sex mismatch (female donor to male recipient) appear to be ideal candidate indications for the use of ATG in related and in unrelated PBSCT.

Discussing the differential effects of ATG in recipients stratified by HLA-C KIR-L status, it is worth emphasizing the importance of separately analyzing transplants using ATG at lower and higher doses, respectively. There is evidence for an increased relapse risk if ATG is used at high doses [7,25,26], while on the other hand, lower-dose ATG was shown not to affect the relapse risk [25,27] and to be associated with improved survival [28,29]. Therefore, it is not surprising that the presented subgroup analyses of lower-dose ATG-treated patients provided particularly favorable survival outcomes. Although it was not the primary endpoint of the present study, it is noteworthy to state that within the respective dose levels (e.g., ATG-F 20–30 mg/kg compared to Thymoglobuline ≤5 mg/kg, or ATG-F ≥40 mg/kg compared to Thymoglobuline ≥7.5 mg/kg) outcomes were similar for both preparations. This suggests that a dose conversion factor for the two rabbit ATGs of ×4 to ×6 may be assumed for “bioequivalence” in HSCT, in accordance with the suggestions by the recently published guidelines on the prevention of GVHD [30].

In C1/1 recipients, comprising the second largest subgroup by KIR-L status among Caucasians, our findings reveal that serotherapy with ATG is associated with a clinically relevant survival benefit. Obviously, this survival benefit associated with ATG among C1/1 recipients results from the reduction of severe aGVHD, translating into reduction of NRM. A protective effect of ATG against moderate/severe or extensive cGVHD may also have contributed to the limitation of NRM in C1/1 recipients, although this effect appeared to require higher ATG doses. Likewise, an increased relapse risk among C1/1 recipients was detected only if higher ATG doses were included in the analysis, suggesting a relevant link between cGVHD and graft versus leukemia (GVL) effects in the C1/1 group. At the bottom line, however, the efficient reduction of NRM at least outweighed the potentially increased relapse risk in C1/1 recipients in the case of higher ATG doses.

In C1/2 recipients, representing the largest subgroup by KIR-L status in Caucasians, ATG was associated with improved OS if applied at lower doses. This benefit did not reach the extent and significance that was revealed for C1/1 recipients. This difference is apparently due to the weaker protective effect of ATG against severe aGVHD in C1/2, as compared to C1/1 recipients. Accordingly, in the C1/2 group, protection from aGVHD-associated NRM and overall NRM was less pronounced than it was in C1/1 recipients. On the other hand, moderate/severe or extensive cGVHD was most effectively limited by ATG in the C1/2 subgroup, even at lower ATG doses. Considering the generally assumed association of cGVHD with GVL effects, one might expect from this finding that ATG would increase the relapse risk particularly in C1/2 recipients. Surprisingly, the opposite was the case; specifically, in C1/2 recipients, the relapse risk remained entirely unaffected by ATG, irrespective of the ATG dose level, and despite its efficient protection against severe cGVHD. The most likely explanation for this unexpected observation is the existence of an effect (possibly NK cell rather than T cell mediated), allowing, to a certain extent, the segregation of GVL from GVHD particularly in C1/2 recipients. This novel hypothesis is further supported by the notion that reduced intensity compared to myeloablative conditioning was associated with a trend toward improved survival in the C1/2 cohort (RR for death, 0.67, for RIC/NMA; *p =* 0.08), suggesting a potent GVL effect timely taking over leukemia control. On the other hand, C1/1 recipients fared significantly worse in the case of reduced intensity conditioning (RR for death, 1.59 for RIC/NMA; *p =* 0.04), implying a weaker GVL effect and therefore stronger dependence on an effective antileukemic conditioning regime. Together, these observations suggest the net GVL effect (i.e., “GVL minus GVHD”) to be greater in the C1/2 than in the C1/1 or the C2/2 cohort, in PBSCT. One might speculate that the presence of both C1 and C2 KIR ligands, a prerequisite for a larger number of “licensed” NK cells, may facilitate NK cell-mediated GVL effects in this cohort, making a contribution of T cells less essential. 

Recipients homozygous for HLA-C2 KIR ligands, in contrast to those with C1/1 or C1/2 KIR ligands, had no survival benefit from ATG at any dose level. Rather, ATG had a detrimental survival effect in this smallest cohort (16% of all HSCT recipients in the present study). For the most part, this was evidently due to an increased relapse incidence associated with ATG. Moreover, despite significant reduction of cGVHD, ATG did not effectively prevent severe aGVHD, and even less, aGVHD-associated NRM. Again, the significantly increased relapse risk in C2/2 donor/recipient pairs may be explained by an impaired NK cell reactivity in this setting, which has been previously reported [20,31,32], and which has been attributed to the inhibitory effect of KIR2DL2/3, recognizing not only C1 but also C2 ligands [33], among other proposed mechanisms. 

As a result of the above findings, recipients with at least one HLA-C1 KIR-ligand, representing 84% of the overall cohort, had a benefit from receiving ATG. C1/1 recipients profited from the vigorous reduction of their otherwise high risk for severe aGVHD and NRM even after matched related transplants. C1/2 recipients benefited because GVL effects remained entirely unaffected by the mechanisms controlling aGVHD and cGVHD. On the other hand, our findings suggest that in C2/2 recipients, serotherapy with ATG should be avoided in HLA-matched PBSCT, since this measure not only failed to reduce NRM, but further escalated the already high [20,31,32] relapse risk. Of note, a recently published study failed to validate an impact of the HLA-C KIR-L status in a large, heterogeneous registry cohort comprising HLA-matched and mismatched BMT and PBSCT, implementing or not implementing anti-T-cell serotherapy [34]. Since no separate analyses are shown for strata by graft source (BMT versus PBSCT) or by the use of ATG (yes vs. no), the authors cannot exclude effects of the HLA-C KIR-L constellation existing within these subgroups. We have previously discussed the necessity of separately analyzing BMT and PBSCT for KIR-L effects, because these effects may be in part opposing after these two different treatment modalities [20]. Moreover, the present findings argue for separate analysis of KIR-L effects in ATG-based versus T cell replete transplantation, because ATG may entirely eliminate some of these effects, such as the high risk for severe aGVHD in C1/1 individuals after HLA-matched, related PBSCT. 

Our findings furthermore indicate that ATG may operate through paving the way toward better manageability, facilitating steroid- (or treatment-) responsiveness of aGVHD, as previously suggested [8], rather than by simply reducing its numerical incidence. This property should be considered for designing studies and declaring endpoints in future (prospective) research on the effects of ATG serotherapy in HSCT.

Except from the recently published “ATG Family Study” [35] and a few other studies [2,7,25,27], patients within most prospective [36,37,38] and the earlier retrospective studies [39,40,41] received ATG mainly in case of an unrelated HSCT. Accordingly, for malignant diseases, many centers use ATG primarily, or exclusively, in the unrelated setting. However, the excess risk for severe aGVHD and cGVHD in matched unrelated, compared to matched sibling transplants has decreased since HLA-matching improved during recent decades [42,43]. Therefore, and in view of the positive results of the “ATG Family Study” [35], we suggest the possible benefits of ATG should not be categorically withheld from recipients of sibling transplants any longer. Instead, risk factors beyond an unrelated or mismatched donor, such as the HLA-C KIR ligand status (C1/1 or C1/2) and a sex-mismatched female donor, may establish as additional indications for the use of ATG in the future. 

Concerning the stem cell source, the benefit of ATG may be more pronounced in peripheral blood HSCT than in BMT, as reflected by the increasing frequency of positive studies showing a survival benefit in the current, PBSCT-dominated era. Indeed, two recent studies revealed a greater benefit of ATG in PBSCT than in BMT [38,44]. An explanation for this finding may be the higher burden of severe cGVHD following PBSCT compared to BMT in case of T cell-replete, ATG-free transplantation. Since the number of BM transplants in the present study is too small for valid subgroup analyses according to KIR-L status, modulation of ATG effects by HLA-C KIR-L can be assumed exclusively for PBSCT at this time. 

As with retrospective, hypothesis-generating studies in general, the present findings should be corroborated by a prospective study randomizing for the use of ATG in KIR-ligand defined strata. Meanwhile, this issue might be elegantly approached by post-hoc stratification for the recipient’s HLA-C KIR-ligand status within randomized studies on ATG that are already completed [35,36,37,38].

The robust findings of the present analysis in the context of a multitude of other studies [2,7,13,14,15,16,17,18,25,27,35] have prompted us to extend the use of ATG to matched-related PBSCT under consideration of the HLA-C KIR-L status. 

## Figures and Tables

**Table 1:** Patient, disease, and treatment characteristics.

Variable	*N* (%)
**Cohort Size, Overall**	775
**Transplant Indication**	
Myeloid malignancies	520 (67.1)
AML/MDS	408
Myeloproliferative and MDS/MPN overlap neoplasia	112
Lymphoid malignancies	228 (29.4)
ALL, including lymphoblastic lymphoma	110
Lymphoma, including CLL	77
Myeloma	41
Non-malignant disorders	27 (3.5)
**Disease Stage at Transplant**	
Early/low-risk	288 (37.2)
Intermediate or advanced/high-risk	487 (62.8)
**Conditioning Regimen**	
Myeloablative (including reduced toxicity myeloablative) *	527 (68.0)
Reduced intensity or non-myeloablative **	248 (32.0)
**Donor Type**	
Sibling	393 (50.7)
HLA matched	388
1 Ag mismatched	5
Unrelated	382 (49.3)
HLA matched (10/10 or 12/12 including DRB3-5)	246
HLA mismatched	136
**Graft Source**	
Bone marrow	143 (18.5)
Peripheral blood (G-CSF mobilized)	632 (81.5)
**Incorporation of ATG**	
No	425 (54.8)
Yes	350 (45.2)
ATG-Fresenius (Grafalon, Neovii)	256
Lower dose ***	160
Higher dose ***	96
Thymoglobuline	87
Lower dose ***	71
Higher dose ***	16
Alemtuzumab ***	7 (0.9)
**Post-grafting Immunosuppression**	
MTX-based (mainly CsA/MTX)	340 (43.9)
MTX-free (mainly CsA/MMF)	435 (56.1)
**HLA-C KIR-L Status**	
C1/1	291 (37.5)
C1/2	363 (46.9)
C2/2	121 (15.6)

* Myeloablative conditioning (MAC) regimens included full intensity MAC (total body irradiation (TBI) 10–13.2 Gy plus high-dose cyclophosphamide ± etoposide, or busulfan 12.8 mg/kg i.v. (or equivalent oral dose) plus high-dose cyclophosphamide ± etoposide), or reduced toxicity myeloablative conditioning (RTC, fludarabine plus i.v. busulfan 9.6–12.8 mg/kg ± thiotepa, or fludarabine plus 8.25–10 Gy TBI). ** Reduced intensity conditioning (RIC) included fludarabine plus either 4 Gy TBI or i.v. busulfan 6.4 mg/kg ± thiotepa. Non-myeloablative (NMA) regimen included fludarabine ± cyclophosphamide ± a maximum dose of either 2 Gy TBI or i.v. busulfan 3.2 mg/kg. In patients with refractory acute leukemia, the preparative regimen was intensified either by the addition of etoposide or thiotepa, or by application of a sequential protocol consisting of a 4-day FLAC (fludarabine, cytarabine) or FLAMSA (fludarabine, cytarabine ± amsacrine) induction phase followed after a 3-day rest by a TBI- or busulfan-based RIC or RTC. *** For anti-thymocyte globulin (ATG)-F, a lower dose was defined as ≤30 mg/kg total, and higher doses were those ≥35 mg/kg total. For Thymoglobuline, a lower dose was defined as ≤5 mg/kg total, and higher doses were those ≥7.5 mg/kg total. Alemtuzumab was dosed 40–100 mg flat (independent of bodyweight), with a median dose of 50 mg. All alemtuzumab doses were assigned to the higher-dose serotherapy group. HLA: human leukocyte antigen; AML, acute myeloid leukemia; MDS, myelodysplastic syndromes; ALL, acute lymphoblastic leukemia; CLL, chronic lymphocytic leukemia; MTX, methotrexate

**Table 2:** Multivariable analyses for overall mortality—entire cohort.

Cohort (*n*) Variable (Reference Group)	Risk Ratio	*p*-Value
**All KIR-L States (*n =* 775)**		
ATG—all dose levels (ref.: no ATG)	0.71	0.014
unrelated donor (ref.: related donor)	1.45	0.015
disease status—int./advanced (ref.: early)	2.17	<0.0001
HLA mismatch (ref.: HLA match)	1.42	0.008
donor age (continuous, per year)	1.01	0.020
MTX-based GVHD prophylaxis (ref.: no MTX)	0.78	0.014
ATG—lower dose levels (ref.: no ATG; *n* = 660)	0.61	0.001
**C1/1 KIR Ligand State (*n =* 291)**		
ATG—all dose levels (ref.: no ATG)	0.50	0.003
disease status—int./advanced (ref.: early)	2.00	0.0001
unrelated donor (ref.: related donor)	1.84	0.008
ATG—lower dose levels (ref.: no ATG; *n* = 237)	0.42	0.001
**C1/2 KIR Ligand State (*n =* 363)**		
ATG—all dose levels (ref.: no ATG)	0.70	0.065
disease status—int./advanced (ref.: early)	2.04	<0.0001
unrelated donor (ref.: related donor)	1.55	0.034
sex mismatch—female to male (ref.: all other)	1.38	0.038
MTX-based GVHD prophylaxis (ref.: no MTX)	0.63	0.008
RIC Regimen (Ref.: myeloablative)	0.71	0.039
ATG—lower dose levels (ref.: no ATG; *n* = 322)	*0.60*	*0.018*
**C2/2 KIR Ligand State (*n =* 121)** Inclusion of all ATG dose levels (121)		
ATG—all dose levels (ref.: no ATG)	1.50	0.17
disease status—int./advanced (ref.: early)	3.46	<0.0001
donor age (continuous, per year)	1.03	0.011
ATG—lower dose levels (ref.: no ATG; *n* = 101)	1.69	0.15

**Table 3:** Multivariable analyses for overall survival, restricted to HLA-identical peripheral blood stem cell transplantation (PBSCT) for hematological malignancies.

Cohort (*n*)/Variable	Risk Ratio	*p*-Value
**All KIR-L States (*n =* 507)**		
ATG—all dose levels (ref.: no ATG)	0.73	0.08
disease status—int./advanced (ref.: early)	2.31	<0.0001
MTX-based GVHD prophylaxis (ref.: no MTX)	0.76	0.04
ATG—lower dose levels (ref.: no ATG; *n* = 449)	0.62	0.02
**C1/1 KIR Ligand State (*n =* 190)**		
ATG—all dose levels (ref.: no ATG)	0.55	0.04
disease status—int./advanced (ref.: early)	2.14	0.0005
ATG—lower dose levels (ref.: no ATG; *n* =160)	0.38	0.005
**C1/2 KIR Ligand State (*n =* 251)**		
ATG—all dose levels (ref.: no ATG)	0.70	0.17
disease status—int./advanced (ref.: early)	2.39	0.0001
MTX-based GVHD prophylaxis (ref.: no MTX)	0.62	0.03
ATG—lower dose levels (ref.: no ATG; *n* = 231)	0.62	0.10
**C2/2 KIR Ligand State (*n =* 66)**		
ATG—all dose levels (ref.: no ATG)	2.59	0.06
Disease status—int./advanced (ref.: early)	4.97	0.0005
unrelated donor	0.32	0.03
ATG—lower dose levels (ref.: no ATG; *n* = 58)	4.99	0.08

**Table 4:** Cumulative incidences of aGVHD III–IV and GVHD-associated NRM according to ATG prophylaxis.

Clinical Endpoint	Entire Cohort *n =* 775	C1/1 *n =* 291	C1/2 *n =* 363	C2/2 *n =* 121
aGVHD III–IV (12 mo)	27.1 (23.2–31.7)	30.6 (24.2–38.6)	26.4 (20.9–33.4)	21.1 (13.5–33.1)
without ATG (%)	*n =* 425	*n =* 161	*n =* 193	*n =* 71
aGVHD III–IV (12 mo)	21.9 (18.0–26.7)	21.1 (15.1–29.5)	23.0 (17.5–30.3)	20.4 (11.8–35.5)
with ATG (%)	*n =* 350	*n =* 130	*n =* 170	*n =* 50
ATG effect by multivariate	RR, 0.63	RR, 0.44	RR, 0.77	RR, 0.49
analysis	*p =* 0.02	*p =* 0.01	*p =* 0.35	*p =* 0.28
GVHD-ass. NRM * (24 mo)	24.9 (21.1–29.4)	28.1 (21.9–36.0)	23.5 (18.2–30.4)	21.2 (13.6–33.3)
without ATG (%)	*n =* 425	*n =* 161	*n =* 193	*n =* 71
GVHD-ass. NRM (24 mo)	20.5 (16.6–25.3)	18.4 (12.5–26.9)	21.2 (15.8–28.5)	24.1 (14.7–39.5)
with ATG (%)	*n =* 350	*n =* 130	*n =* 170	*n =* 50
ATG effect by multivariate	RR, 0.51	RR, 0.28	RR, 0.55	RR, 1.34
analysis	*p =* 0.001	*p =* 0.0002	*p =* 0.04	*p =* 0.6

* Defined as any non-relapse mortality within 24 months after transplant in a patient with active or previous aGVHD requiring systemic treatment (i.e., grade 2–4).
